# The high-quality genome of *Grona styracifolia* uncovers the genomic mechanism of high levels of schaftoside, a promising drug candidate for treatment of COVID-19

**DOI:** 10.1093/hr/uhae089

**Published:** 2024-03-30

**Authors:** Shaohua Zeng, Zhiqiang Wang, Dingding Shi, Fangqin Yu, Ting Liu, Ting Peng, Guiqi Bi, Jianbin Yan, Ying Wang

**Affiliations:** Guangdong Provincial Key Laboratory of Applied Botany, Key Laboratory of National Forestry and Grassland Administration on Plant Conservation and Utilization in Southern China, South China National Botanical Garden, State Key Laboratory of Plant Diversity and Specialty Crops, South China Botanical Garden, Chinese Academy of Sciences, Guangzhou 510650, China; College of Life Sciences, Gannan Normal University, Ganzhou 341000, China; University of Chinese Academy of Sciences, Beijing 100049, China; Guangdong Provincial Key Laboratory of Applied Botany, Key Laboratory of National Forestry and Grassland Administration on Plant Conservation and Utilization in Southern China, South China National Botanical Garden, State Key Laboratory of Plant Diversity and Specialty Crops, South China Botanical Garden, Chinese Academy of Sciences, Guangzhou 510650, China; University of Chinese Academy of Sciences, Beijing 100049, China; Guangdong Provincial Key Laboratory of Applied Botany, Key Laboratory of National Forestry and Grassland Administration on Plant Conservation and Utilization in Southern China, South China National Botanical Garden, State Key Laboratory of Plant Diversity and Specialty Crops, South China Botanical Garden, Chinese Academy of Sciences, Guangzhou 510650, China; University of Chinese Academy of Sciences, Beijing 100049, China; Guangdong Provincial Key Laboratory of Applied Botany, Key Laboratory of National Forestry and Grassland Administration on Plant Conservation and Utilization in Southern China, South China National Botanical Garden, State Key Laboratory of Plant Diversity and Specialty Crops, South China Botanical Garden, Chinese Academy of Sciences, Guangzhou 510650, China; College of Life Sciences, Gannan Normal University, Ganzhou 341000, China; Guangdong Provincial Key Laboratory of Applied Botany, Key Laboratory of National Forestry and Grassland Administration on Plant Conservation and Utilization in Southern China, South China National Botanical Garden, State Key Laboratory of Plant Diversity and Specialty Crops, South China Botanical Garden, Chinese Academy of Sciences, Guangzhou 510650, China; College of Life Sciences, Gannan Normal University, Ganzhou 341000, China; College of Life Sciences, Gannan Normal University, Ganzhou 341000, China; Shenzhen Branch, Guangdong Laboratory of Lingnan Modern Agriculture, Key Laboratory of Synthetic Biology, Ministry of Agriculture and Rural Affairs, Agricultural Genomics Institute at Shenzhen, Chinese Academy of Agricultural Sciences, Shenzhen 518124, China; Shenzhen Branch, Guangdong Laboratory of Lingnan Modern Agriculture, Key Laboratory of Synthetic Biology, Ministry of Agriculture and Rural Affairs, Agricultural Genomics Institute at Shenzhen, Chinese Academy of Agricultural Sciences, Shenzhen 518124, China; Guangdong Provincial Key Laboratory of Applied Botany, Key Laboratory of National Forestry and Grassland Administration on Plant Conservation and Utilization in Southern China, South China National Botanical Garden, State Key Laboratory of Plant Diversity and Specialty Crops, South China Botanical Garden, Chinese Academy of Sciences, Guangzhou 510650, China; College of Life Sciences, Gannan Normal University, Ganzhou 341000, China; University of Chinese Academy of Sciences, Beijing 100049, China

## Abstract

Recent study has evidenced that traditional Chinese medicinal (TCM) plant-derived schaftoside shows promise as a potential drug candidate for COVID-19 treatment. However, the biosynthetic pathway of schaftoside in TCM plants remains unknown. In this study, the genome of the TCM herb *Grona styracifolia* (Osbeck) H.Ohashi & K.Ohashi (GSO), which is rich in schaftoside, was sequenced, and a high-quality assembly of GSO genome was obtained. Our findings revealed that GSO did not undergo recent whole genome duplication (WGD) but shared an ancestral papilionoid polyploidy event, leading to the gene expansion of chalcone synthase (*CHS*) and isoflavone 2′-hydroxylase (*HIDH*). Furthermore, GSO-specific tandem gene duplication resulted in the gene expansion of C-glucosyltransferase (*CGT*). Integrative analysis of the metabolome and transcriptome identified 13 *CGTs* and eight *HIDHs* involved in the biosynthetic pathway of schaftoside. Functional studies indicated that *CGTs* and *HIDHs* identified here are *bona fide* responsible for the biosynthesis of schaftoside in GSO, as confirmed through hairy root transgenic system and *in vitro* enzyme activity assay. Taken together, the ancestral papilionoid polyploidy event expanding *CHSs* and *HIDHs*, along with the GSO-specific tandem duplication of CGT, contributes, partially if not completely, to the robust biosynthesis of schaftoside in GSO. These findings provide insights into the genomic mechanisms underlying the abundant biosynthesis of schaftoside in GSO, highlighting the potential of GSO as a source of bioactive compounds for pharmaceutical development.

## Introduction

**Figure 1:**
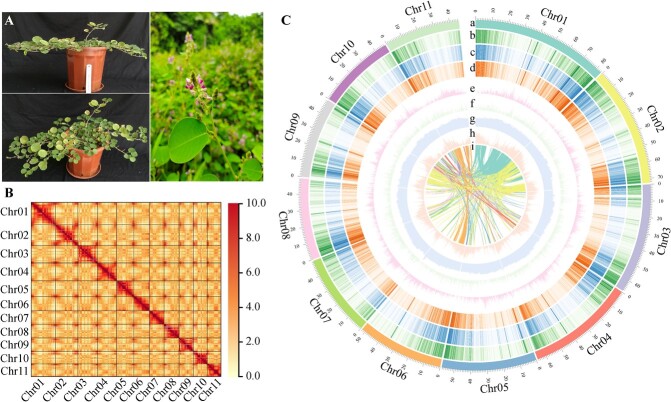
The GSO plants and its genome landscape. **A** the phenotype of GSO. **B** intensity heat map of Hi-C chromosome interaction for GSO; The high probabilities of contact is denoted by red yellow pixels. Most interactions were observed within the chromosomes. **C** the genome landscape of GSO. a, pseudochromosomes number and length (on a Mb scale); b–d, the gene expression level at 10ML/10MS/10MR, separately; e, distribution of Gypsy-type transposons (sliding window size 100 Kb); f, distribution of Copia-type transposons (sliding window size 100 Kb); g, the density of genes; h, the distribution of GC content (sliding window size 100 Kb); i, genome synteny.

Recently, it has been reported that traditional Chinese medicine has demonstrated efficacy in treating coronavirus disease 2019 (COVID-19) caused by severe acute respiratory syndrome coronavirus 2 (SARS-CoV-2) [1]. Recent study has also evidenced that schaftoside inhibits the 3CL^pro^ and PL^pro^ of SARS-CoV-2 virus, while also enhancing the immune response of host cells treated by COVID-19. This dual mechanism positions schaftoside as a promising candidate for the treatment of COVID-19 [2]. In 2022, the total flavonoids capsule derived from Herba *Desmodii styracifolii*, an innovative traditional Chinese medicine, received approval from the National Medical Products Administration of China (Z20220003). According to the Chinese pharmacopoeia, schaftoside is the characteristic compound of Herba *Desmodii styracifolii*, which is obtained from the dried aerial parts of the *Desmodium styracifolium* Merr. plant. This herb is known for its significant therapeutic effects on the conditions such as urination disturbance, urolithiasis, edema, and jaundice [3]. Recently, *D. styracifolium* was taxonomically renamed as *Grona styracifolia* (Osbeck) H.Ohashi & K.Ohashi, hereafter referred to as GSO (Fig. 1A) [4]. GSO contains abundant terpenoids and flavonoids, which are primarily responsible for its pharmacological properties. The key flavonoids present in GSO are schaftoside (Apigenin-6C-Glucoside-8C-Arabinoside) and isoschaftoside (Apigenin-6C-Arabinoside-8C-Glucoside), which exhibit diverse biological activities, including anti-respiratory syncytial virus, antidiabetic properties, antihypertensive effects, hepatoprotective benefits, and anti-inflammatory actions [5]. Furthermore, schaftoside has been shown to have positive effects in various conditions, such as treating pentylenetetrazol-induced seizures [6], exerting anti-melanogenic activity [7], alleviating nonalcoholic fatty liver disease induced by a high-fat diet [8], and restoring renal function [9].

Although the biosynthesis of aglycone apigenin has been extensively studied, little is known about the modification/decoration of apigenin derivatives such as schaftoside, which was recently predicted in the related plant *Desmodium spp.* [10]. Furthermore, the C-glycosyltransferase enzymes responsible for biosynthesizing C-glycosylated flavonoids have been characterized [5]. However, the exact biosynthetic pathway of schaftoside in GSO remains unknown. Additionally, the genomic mechanisms underlying the robust biosynthesis of schaftoside in GSO are not well understood. Recently, whole-genome sequencing technology has been successfully employed to study the specialized metabolites in medicinal plants such as *Astragalus sinicus* [11], *Cercis chinensis* [12], and *Morinda officinalis* [13]. Genomic information serves as a foundation for identifying functional genes, facilitating the molecular breeding, and improving the quality of medicinal materials [14]. However, the genome sequence of GSO has been not yet reported to date, which severely restricts the functional genomics and molecular breeding in GSO.

In this study, the genome of GSO was sequenced and *de novo* assembled through a combination of advanced technologies to elucidate its genomic characteristics, including PacBio sequencing, Illumina sequencing, and Hi-C technology. Comparative Genomics with other sequenced legume species, along with integrative analysis of transcriptome and metabolome data, were performed to obtain fundamental insights into the genetic mechanisms underlying flavonoids biosynthesis, particularly schaftoside production. Our findings reveal that GSO did not undergo recent whole genome duplication (WGD) but experienced an ancestral papilionoid polyploidy event, leading to the gene expansion of chalcone synthase (*CHS*) and isoflavone 2′-hydroxylase (*HIDH*). GSO-specific tandem gene duplication resulted in the gene expansion of C-glucosyltransferase (*CGT*), which resulting in the robust biosynthesis of schaftoside in GSO. The reference genome of GSO obtained here will serve as a valuable resource for elucidating the biosynthesis of bioactive ingredients and facilitating the genetic improvement of *G. styracifolia*.

## Results

### Genome sequencing and annotation

In this study, the GSO genome was sequenced and *de novo* assembled using a combination of technologies to elucidate its genomic characteristics, including PacBio CLR sequencing, Illumina sequencing, and Hi-C sequencing (Table S1, see online supplementary material). Genome survey indicates that the estimated genome size of GSO is 638.82 Mb, with a heterozygosity rate of 0.152% and a transposable elements (TEs) ratio of 72.13% (Fig. S1, see online supplementary material). Flow cytometry estimated the genome size to be 581.2 Mb (Table S2, Fig. S2, see online supplementary material). The resulting GSO assembly consisted of 11 chromosomes with a genome size of 641.82 Mb and an N50 contig/scaffold of 14.28/57.49 Mb (Fig. 1B and C;Table S3, see online supplementary material). It seems that the genome size of GSO is slightly larger than its derivatives common bean (*Phaseolus vulgaris* L., 587 Mb) [50] and mung bean (*Vigna radiate*, 579 Mb) [51], which might be attributed to the higher ratio of TEs in GSO genome when compared to *P. vulgaris* (45.4%) and *V. radiate* (50.1%). Based on the combined data from *ab initio*, homology-based analyses, and RNA sequencing-assisted annotation, the GSO genome contains 35 865 protein-coding genes (Table S4 and Fig. S3, see online supplementary material), which is significantly higher than *P. vulgaris* (27197) and *V. radiate* (22427) [50, 51].

**Figure 2:**
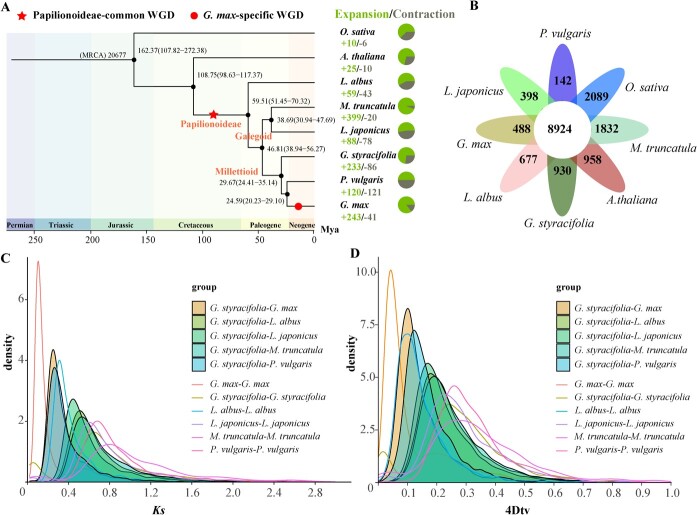
Phylogenetic and evolutionary analysis of GSO. **A** Divergence time estimation and gene family expansion/contraction analyses. The numbers on the nodes represent the divergence time of the species (million years ago, Mya), with confidence range in brackets. The gain (expansion) and loss (contraction) number of gene families were indicated by green and grey pies, respectively. The red star indicates the whole genome duplication event shared by the legume species. The orange circle indicates the tandem duplication in GSO. **B** Distributions of synonymous substitutions per site (*Ks*) of syntenic blocks of GSO paralogs and orthologs with other eudicots. **C** Four-fold synonymous (degenerative) third-codon transversion (4Dtv) of one-to-one orthologs identified between GSO, *Glycine max*, *Lupinus albus*, *Lotus japonicus*, *Medicago truncatula*, and *Phaseolus vulgaris*. **D** Venn analysis of common and species-specific gene families in GSO genome.

The quality of the assembled genomes was assessed through various analyses. Firstly, the high LTR assembly index (LAI) score of 19.0 indicated that the continuity of the GSO genome approached to reference quality (Fig. S4, see online supplementary material). Secondly, 93.5% to 97.8% of the Illumina reads successfully mapped to the genome assembly, supporting a high level of genome coverage (Table S5, see online supplementary material). Thirdly, a total of 98.6% (1365) of core genes was identified in the GSO genome using the Core Eukaryotic Genes Mapping Approach (CEGMA) analysis (Table S6, see online supplementary material). Furthermore, the Benchmarking Universal Single-Copy Orthologs (BUSCO) assessment indicated that 98.2% and 94.8% of BUSCO gene models for genome assembly and predicted coding genes were respectively identified, suggesting the near completeness of genome assembly and annotation (Table S7, see online supplementary material). These findings demonstrate the high quality of the GSO genome assembly and annotation.

### Genome duplication and evolution analysis

To investigate the evolution of GSO in the legume family, Arabidopsis, rice, and five sequenced legume species, including *Glycine max*, *Medicago truncatula*, *Lupinus albus*, *Lotus japonicus*, and *P. vulgaris* were compared, which resulted in the identification of 28 193 gene families and 658 single-copy genes (Fig. 2A; Figs S5 and S6, see online supplementary material). Maximum-likelihood phylogenetic analysis using the single-copy genes revealed that GSO is closely related to *G. max* and *P. vulgaris*. MCMCTree analysis showed that *P. vulgaris* and *G. max* diverged at 24.59 million years ago (MYA), which is similar to the previous estimation [50], and that the palaeopolyploidy event shared by all bean-like (papilionoid) legume species occurred at ~65 MYA, which is consistent to previous studies [52]. According to the MCMCTree analysis, GSO and *G. max* diverged at approximately 29.67 MYA (Fig. 2A).

Whole genome duplication (WGD) is an important evolutionary force contributing to the diversity of specialized metabolites in plants [53]. In addition, WGD is a ubiquitous feature and an evolutionary driver of genetic innovations in flowering plants [54, 55]. Synonymous substitutions per site (*Ks*) and 4-fold synonymous third-codon transversion (4DTv) rates of the duplicated gene pairs were investigated to assess the occurrence of WGD events in GSO. The genome synteny and *Ks* values of paralogs and orthologs among GSO, *G. max*, *M. truncatula*, and *Vitis vinifera* genomes were investigated.

The intra-genomic collinearity showed that the GSO experienced only one WGD event (Papilionoideae-common WGD) after the core-eudicot whole-genome triplication (WGT, or γ event) (Fig. S7, see online supplementary material). In contrast, the *G. max* underwent two rounds of polyploidization (Gm-beta and Gm-alpha genome duplications) (Fig. S7, see online supplementary material) [56, 57]. The median *Ks* value of paralog gene pairs on synteny blocks from the GSO genome was about 0.75, while the Gm-beta and Gm-alpha corresponded to the median *Ks* values of ~0.1 and ~ 0.75, indicating they may share a WGD events (Figs S7 and S8, see online supplementary material). Subsequently, the well-characterized grape (*V. vinifera*) genome, which is a relatively stable genome and is likely not affected by any polyploidization event after the γ event [58], was used as a reference for inter-genomic dotplot comparison with GSO and *G. max*. If there had been an extra diploidization event in GSO, assuming no DNA loss, we would expect a grape gene (or chromosomal region) to have four best-matched or orthologous GSO genes (chromosomal regions). However, the ratios of the best-matched orthologous regions between two species (GSO and *G. max*) and *V. vinifera* were 2:1 and 4:1, respectively (Figs S9–S11, see online supplementary material). Moreover, *G. max* and *M. truncatula* compared with GSO show a 2:1 and 1:1 result, respectively (Figs S12 and S13, see online supplementary material). In summary, our findings suggest that GSO did not experience a recent WGD but instead shared a papilionoideae WGD event described in a previous study [59] (Fig. 2C and D).

### Gene family analysis

Positive selection is a critical driving force for gene neofunctionalization in species [60, 61]. To evaluate the positive selection in GSO, the Ka/Ks ratio of the single-copy genes was calculated. A total of 82 genes undergo positive selection (*P*  < 0.05) and are over-represented in gene ontology (GO) terms related to ‘meiotic chromosome segregation’, ‘DNA topoisomerase’, ‘DNA repair’, and ‘homologous recombination’ (Fig. S14 and Tables S8–S10, see online supplementary material). OrthoMCL analysis indicated that a total of 32 633 (91.0%) genes were categorized into 17 607 gene families, with 930 of them being specific to GSO (Fig. 2B; Table S11, see online supplementary material). As shown in Fig. S15 and Tables S12 and S13 (see online supplementary material), the KEGG pathway related to the indeterminate nodule organogenesis, including ‘cell cycle’, ‘DNA replication’, ‘Plant-pathogen interaction’, and ‘NOD-like receptor signaling pathway’ are over-represented, which agree with previous study [11]. Notably, ‘Flavonoid biosynthesis’ and ‘flavone and flavonol biosynthesis’ are also enriched. These results suggest that the evolutionarily generated species-specific gene families contribute to active nodule organogenesis and flavonoid production in GSO.

**Figure 3:**
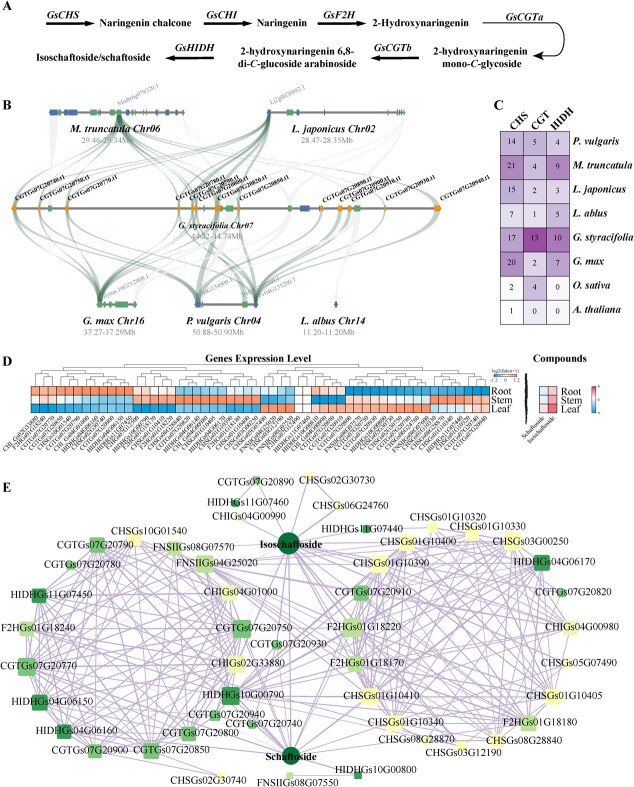
The biosynthesis of schaftoside in GSO. **A** the biosynthetic pathway of schaftoside. **B** The collinear relationship of *CGT* in GSO, *Glycine max*, *Medicago truncatula*, *Lupinus albus*, *Lotus japonicus*, and *Phaseolus vulgaris*. The orange box indicated *GsCGTs* in GSO. The forward and reverse directions of genes on chromosomes are labeled with green and blue box, respectively. The syntenic blocks are connected by light-green lines. **C** The number of *CHS* (chalcone synthase), *CGT* (C-glycosyltransferase), and *HIDH* (2-hydroxyisoflavanone dehydratase) in GSO and other seven species. **D** The expression profile of schaftoside and its predicted biosynthetic genes in roots, stems, and leaves. **E** The co-expression network of schaftoside and biosynthetic genes. The shape size is proportional to the number of nodes linked, and the line thickness is proportional to the size of the correlation.

The CAFE analysis revealed that GSO has expanded 233 gene families and contracted 86 gene families (Fig. 2A). KEGG enrichment analysis of the expanded genes showed an overrepresentation of pathways such as ‘DNA replication’, ‘nucleotide excision repair’, ‘homologous recombination’, ‘mismatch repair’, ‘plant-pathogen interaction’, ‘phenylpropanoid biosynthesis’, ‘anthocyanin biosynthesis’, and ‘isoflavonoid biosynthesis’ (Fig. S16 and Table S14, see online supplementary material). These results suggest a significant expansion of genes related to secondary metabolic pathways, particularly nodule organogenesis-related flavonoid genes. GO enrichment analysis of the expanded genes indicated involvement in processes such as ‘flavonoid metabolic process (18 genes)’, ‘UDP-glycosyltransferase activity (UGT, 34 genes)’, O-methyltransferase activity (31 genes), and ‘monooxygenase activity (83 genes)’ (Fig. S16 and Table S15, see online supplementary material). Notably, among the expanded UGTs, a cluster of 13 UGTs belonging to the UGT708 clade encoding C-glucosyltransferase (CGT) was found on chromosome 7 (Chr7) (Fig. S17, see online supplementary material). Previous studies documented that CGT is involved in C-glycosylation of flavonoids [62, 63]. These findings suggest that the expanded CGTs in GSO may contribute to the high levels and diversity of flavonoid C-glucosides. Among the expanded O-methyltransferase genes, 10 genes encode isoflavone-7-O-methyltransferase (IF7OMT) clustered on Chr4. Similarly, 21 out of 83 expanded monooxygenase genes encode isoflavone 2′-hydroxylase (I2’H). Additionally, 10 copies of 2-hydroxyisoflavanone dehydratase (HIDH) were also expanded (Fig. 3). The GSO assembly contains 17 chalcone synthase (CHS) gene members, confirming the expansion of the CHS gene family in legume species [11]. These findings support the hypothesis that an ancestral polyploidy event led to the expansion of flavonoid biosynthetic genes, including *CHS*, *HIDH*, *I2’H*, and *IF70MT*, in the papilionoideae [59]. This conclusion is further supported by the gene number, phylogenetic tree, and collinear analysis in legume species (Figs S18–S24, see online supplementary material). As mentioned above, the GSO genome did not undergo a recent WGD event. Taken together, our findings suggest that the papilionoideae WGD event, leading to the expansion of *CHS* and *HIDH* genes, and the GSO-specific CGT tandem duplication contribute to the robust biosynthesis of flavonoids in GSO, especially for schaftoside.

### Uncovering the schaftoside biosynthetic pathway in GSO

To dissect the schaftoside biosynthesis in GSO, a potential pathway for schaftoside is proposed based on previous studies [5, 10], which include 17 *CHSs*, five *CHIs*, six *F2Hs*, 13 *CGTs*, and 10 *HIDHs* structural genes in GSO assembly (Fig. 3A). To further elucidate the genomic mechanism behind the abundant biosynthesis of schaftoside in GSO, the synteny analysis was performed. As shown in Fig. 3B and Figs S22 and S25 (see online supplementary material), *CGT*, *CHS*, and *HIDH* undergo tandem gene duplication events in GSO, resulting in a higher number of *CGTs* in GSO compared to other legume species (Fig. 3C). The diverse expression profile of *CHS*, *CGT*, and *HIDH* members suggest the neo/subfunctionalizational role of these duplicated genes in the biosynthesis of schaftoside (Fig. 3D). The metabolome and transcriptome of eight-month-old roots stems, and leaves are integrated to identify candidate *CGT* and *HIDH* genes responsible for schaftoside biosynthesis. A total of 10 251 DEGs and seven metabolites involved in the biosynthetic pathway of (iso)schaftoside were selected for WGNCA analysis. As shown in Fig. S26 (see online supplementary material), the MEblue and MEturquoise modules were found to be significantly associated with (iso)schaftoside biosynthesis compared to other modules. WGCNA analysis indicated that 13 *CGTs* and eight *HIDHs*, located within the MEblue or MEturquoise modules, are involved in the biosynthetic network of schaftoside (Fig. 3E; Fig. S25, see online supplementary material).

**Figure 4:**
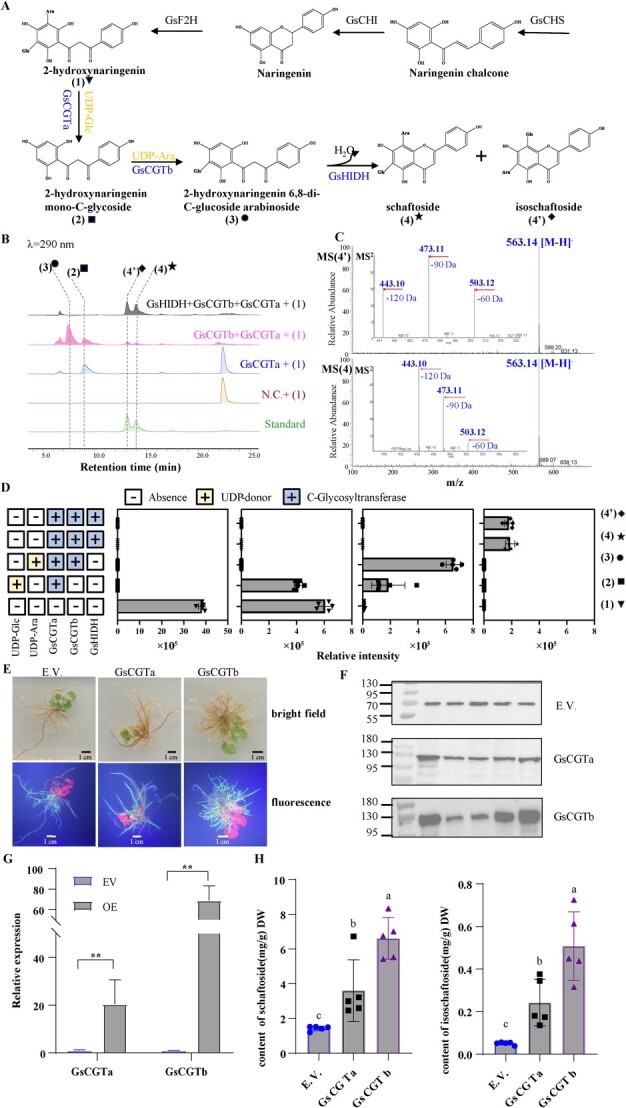
Functional characterization of GsCGTa, GsCGTb, and GsHIDH involved in biosynthesis of schaftoside (4) and isoschaftoside (4′). **A** The corresponding biosynthetic pathway for the stepwise formation of (4) and (4′). CHS, chalcone synthase; CHI, chalcone isomerase; CGT, C-glycosyltransferase; F2H, favanone 2-hydroxylase; HIDH, 2-hydroxyisoflavanone dehydratase. **B** The *in vitro* enzymes activity assay of GsCGTa, GsCGTb, and GsHIDH. Recombinant GsCGTa, GsCGTb, and GsHIDH enzyme produced in *Escherichia coli* cells with 2-hydroxynaringenin (1) as substrate leads to stepwise transformation into (4) and (4′). **C** (−)-ESI-MS and MS [2] spectra of (4) and (4′). **D** The relative intensity of the verified pathway intermediates/products observed upon the addition of GsCGTa, GsCGTb, and GsHIDH enzymes, as measured by HPLC analysis. **E** Positive hairy roots transgened by empty vector (EV) and GsCGT construct with GFP marker were detected under bright field and fluorescence. **F** Transcript levels of *GsCGTs* in EV and in overexpression (OE) lines were detected using qPCR. **G** Relative protein abundance of GsCGTs in EV and OE lines. **H** Relative content of schaftoside and isoschaftoside in EV and OE lines. Data are presented as mean values ± SD (*n* = 5 independent biological replicates). Significant differences (*P*-values <0.05) between the groups were showed with Student’s *t*-test in F or variance (ANOVA) combined with Duncan’s multiple range test.

To validate the function of candidate genes and understand the biosynthetic pathway of schaftoside in GSO, *CGTa*(Gs07G20770), *CGTb*(Gs07G20750), and *HIDH*(Gs10G00790) were selected for metabolic engineering in *Escherichia coli* for functional characterization according to the WGCNA results. As shown in Fig. 4A and B, overexpression of *CGTa*(Gs07G20770), or *CGTa*(Gs07G20770) coupled with *CGTb*(Gs07G20750), produces C-glucosyl-2-hydroxynaringenin and C-glucosyl-C-arabinosyl-2-hydroxynaringenin, respectively, when 2-hydroxynaringenin is used as substrate. Additionally, coexpression of *CGTa*(Gs07G20770), *CGTb*(Gs07G20750), and *HIDH*(Gs10G00790) in *E. coli* successfully produces (iso)schaftoside (Fig. 4B and C). Furthermore, overexpression of *CGTa*(Gs07G20770) or *CGTb*(Gs07G20750) in GSO hairy roots also enhances the content of (iso)schaftoside (Fig. 4E and H). These results confirm that the *CGTs* and *HIDHs* identified in this study are *bona fide* involved in the biosynthesis of schaftoside in *G. styracifolia*.

## Discussion

*G. styracifolia* is a renowned traditional medicinal plant recognized for its high content of schaftoside, which is the characteristic bioactive component recorded in Chinese pharmacopoeia for its therapeutic effects such as urination disturbances, urolithiasis, and jaundice. Recently, schaftoside has also been discovered to possess potential in the treatment of COVID-19 [2]. Although *G. styracifolia* biosynthesizes and accumulates a large amount of schaftoside, the underlying genomic mechanism responsible for its biosynthesis remains elusive. The advent of third-generation high-throughput sequencing has greatly enhanced the ability to obtain high-quality genomes, as demonstrated in species like *A. sinicus* [11], *C. chinensis* [12], and *Magnolia biondii* [13], enabling a deeper understanding of the biosynthesis and regulation of specialized metabolites in these plants. In this study, a comprehensive approach was employed to sequence the genome of *G. styracifolia*, resulting in a near-complete genome assembly with 19.0 LAI, 98.6% CEGMA, and 98.2% BUSCO completeness.

The genomic analysis of GSO revealed an expansion of 233 gene families and a contraction of 86 gene families (Fig. 2A). Particularly, there was enrichment observed in flavonoid-related biosynthetic pathways, potentially contributing to the high levels of flavonoids such as schaftoside. Moreover, 34 UDP-glycosyltransferase genes were found to be overrepresented, with 13 of them encoding UGT708 subclade CGTs arranged in tandem on Chr7. Interestingly, WGD analysis indicated that the GSO assembly did not undergo a recent genome duplication event. This suggests that the gene cluster containing the 13 UGT708 genes likely arose from a tandem duplication event rather than a WGD event. Additionally, the common papilionoideae genome duplication event was found to have contributed to the expansion of *CHS* and *HIDH* genes in the GSO assembly, consistent with a previous study [59]. Furthermore, functional characterization studies confirmed the involvement of *CGTa* (Gs07G20770), *CGTb* (Gs07G20750), and *HIDH* (Gs10G00790) in the biosynthesis of schaftoside in *G. styracifolia*. As a result, the biosynthetic pathway of schaftoside in *G. styracifolia* has been completely elucidated in this study.

In conclusion, a high-quality GSO assembly was obtained, which did not undergo a recent genome duplication event but shared an ancestral papilionoid polyploidy event, leading to the expansion of *CHS* and *HIDH*. Additionally, a GSO-specific tandem gene duplication event resulted in the *CGT* expansion in the GSO genome. Taken together, the ancestral papilionoid polyploidy event expanding *CHSs* and *HIDHs*, along with the GSO-specific tandem duplication of *CGT*, contributes, at least partially, to the robust biosynthesis of schaftoside in GSO.

## Materials and methods

### Plant materials and genome-survey analysis

Young leaves were harvested from seedlings of *G. styracifolia* grown at the South China Botanical Garden, Chinese Academy of Sciences, for the purpose of isolating genomic DNA. The isolated DNA was subsequently utilized for Illumina, PacBio, and Hi-C sequencing. A total of 93.33 gigabase (Gb) of clean paired-end reads were obtained through sequencing on the Illumina HiSeq 6000 platform (Illumina, San Diego, CA, USA). These reads were then subjected to filtering using SOAPnuke software v2.0.2 [15]. Genome survey analysis was conducted by examining *K*-*mer* distribution (*K* = 17) with Jellyfish (v2.3.0) [16] and GenomeScope [17] software to predict genome size, heterozygosity, and repeat-sequence characteristics.

### Genome sequencing, assembly, and quality assessment

The subreads obtained through PacBio sequencing were assembled into contigs using NextDenovo (v2.5.2) (https://github.com/Nextomics/NextPolish). The resulting consensus genome was refined by aligning it to the PacBio subreads using minimap2 (v2.24) [18] and undergoing three rounds of corrections with Racon (v1.4.21) [19]. Subsequently, the 10X Genomics data was mapped to the assembled genome using BWA-MEM (v0.7.17) [20] with default parameters. FragScaff (https://github.com/adeylab/fragScaff) was employed for scaffolding. In addition, Illumina reads were utilized to improve the GSO assembly using Pilon (v1.23) [21]. Chromosome-level assembly was facilitated using Hi-C technology through Juicer (v2.041) [22] and 3D-DNA (v180922) [23]. The final assembly was assessed using BUSCO v5.1.2 [24] (fabales_odb10), CEGMA [25] and LAI (LTR Assembly Index) [26]. The multi-omics data has been deposited in National Genomics Database Center (NGDC, https://ngdc.cncb.ac.cn/?lang=zh) under the accession number PRJCA016945.

### Genome annotation

The repeat sequence structures within the GSO genome were identified and masked using RepeatModeler (http://www.repeatmasker.org/RepeatModeler.html) and RepeatMasker (http://www.repeatmasker.org, v3.3.0), respectively. A comprehensive approach was then employed for gene structure annotation, integrating homology-based, *de novo* gene prediction, and transcriptome-based prediction methods. Initially, homologous proteins from *Arabidopsis thaliana*, *Oryza sativa*, *Glycine max*, and *Phaseolus vulgaris* were aligned to the masked GSO genome using TblastN [27] with an E-value threshold of 1e-5. Subsequently, the BLAST hits were merged using Solar [28]. GeneWise (https://www.ebi.ac.uk/Tools/psa/genewise) was utilized for precise gene structure prediction. Next, GeneMark-ET and AUGUSTUS, integrated within the BRAKER2 framework [29], were employed for *ab initio* gene prediction. RNA-seq reads aligned to the masked genome by HISAT2 [30] were assembled to predict transcripts using Stringtie [31]. The results from the aforementioned methods were integrated using EVidenceModeler (EVM) (http://evidencemodeler.sourceforge.net/) to generate a non-redundant set of gene structures [32], which were further refined and updated by PASA. For gene annotation, the resulting genes sets were compared against several databases, including SwissProt, NR, InterPro (http://www.ebi.ac.uk/interpro/, V32.0), Pfam (http://pfam.xfam.org/, V27.0), InterProScan (V4.8), HMMER (http://www.hmmer.org/, V3.1), GO (http://www.geneontology.org/page/go-database), and KEGG (http://www.kegg.jp/kegg/kegg1.html, release 53) databases based on sequence similarity and domain conservation.

### Comparative genomics analyses

In order to investigate the evolutionary relationship of the GSO genome among legume species, protein sequences from five leguminous species, namely *G. max*, *L. albus*, *L. japonicus*, *M. truncatula*, and *P. vulgaris* were selected for analysis. In addition, two model plants, *A. thaliana* and *O. sativa*, were included as outgroup species. The protein families were classified using OrthoFinder v2.5.1 [33] through a blast analysis with default parameters in diamond. Subsequently, the gene families were annotated using the Pfam 33.1 database. Single-copy gene families were aligned and identified using MAFFT v7.205 [34] and Gblocks v0.91b [35]. The well-aligned gene family sequences for each species were concatenated end-to-end to create supergenes, and the alignments of each gene family were combined into a super-alignment matrix. A maximum likelihood (ML) phylogenetic tree was constructed using RAxML (http://sco.h-its.org/exelixis/web/software/raxml/index.html) with the optimal model determined by ModelFinder [36] implemented in IQ-TREE [37]. The MCMCTree program (http://abacus.gene.ucl.ac.uk/software/paml.html), part of the Phylogenetic Analysis by Maximum Likelihood (PAML) [38], was employed to infer divergence times based on the phylogenetic tree, with calibration points selected from the TimeTree database (http://timetree.org) for *A. thaliana* and *O. sativa*. The CAFE 4.2 (Computational Analysis of gene Family Evolution) program [39] was utilized to evaluate the expansion and contraction of gene families relative to their ancestors. Additionally, the CodeML program in PAML was applied to identify genes under positive selection. Finally, GO and KEGG enrichment analysis were conducted using ClusterProfiler v4.0 [40] for the expanded, contracted, and positively selected genes, respectively.

### Genome synteny and whole-genome duplication (WGD) analysis

To identify syntenic blocks across different species, the protein sequences of *G. max*, *L. albus*, *L. japonicus*, *M. truncatula*, *P. vulgaris*, *A. thaliana*, and *O. sativa* were subjected to self and cross-species searches using DIAMOND v0.9.29.130 [41]. Syntenic block regions containing a minimum of five gene pairs were defined using MCScanX [42]. The *Ks* (synonymous mutation rate) and 4DTv (fourfold synonymous third-codon transversion rate) methods were employed to detect whole-genome duplication events (WGDs). JCVI v0.9.13 was utilized for visualizing synteny blocks between chromosomes. Additionally, wgdi [43] was used to validate the occurrence of WGD events in the GSO species.

### Transcriptome and metabolism analysis

Total RNA was extracted from various tissues of GSO, including the root, stem, leaf, and flower, to construct transcriptome sequencing libraries. The alignment of reads filtered by fastp v0.23.2 [44] to the GSO genome was performed using HISAT2 [30]. Subsequently, SAMtools [45] was employed to convert the alignment results to bam format, and featureCounts [46] was used to generate the reads count matrix. Differential expression analysis was carried out using DESeq2 [47], with the criteria of *P-*adj < 0.05 and |Log2FC| > = 1 for identifying differentially expressed genes (DEGs). Metabolome analysis was conducted on the root, stem, and leaf tissues using LC–MS/MS method as described in a previous study [48]. Differential accumulation of metabolites (DAMs) was identified using the OPLS-DA method, with the criteria of VIP > = 1 and |Log2FC| > = 1. The weighted gene co-expression network analysis (WGCNA) strategy employed in this study followed a previous study [49]. In brief, after filtering out genes with low expression (average FPKM <1), DEGs with a coefficient of variation (CV) > 0.5 were selected for co-expression network module generation using the WGCNA package in R. The co-expression modules were constructed using the blockwiseModules function with default parameters, a soft-threshold power of 2, TOMtype set to signed, mergeCutHeight of 0.25, and minModuleSize of 30. Seven metabolites involved in the (iso)schaftoside biosynthetic pathway were selected for WGCNA analysis in this study.

### Expression and purification of GsCGTs and GsHIDH in *E. coli*

Total RNA was extracted from the stem, root, and leaf tissues of GSO using the HiPure Total RNA Plus Mini Kit (Magen, China). The extracted RNA was then utilized to synthesize first-strand cDNA with the PrimeScript™ II 1st Strand cDNA Synthesis Kit (Takara, Japan). The genes GsCGTs or GsHIDH were integrated into the Nde I/Hind III cloning sites of the pCold II vector (Takara, Japan) via homologous recombination using the Fast DNA Assembly Mix kit (#E0201L) from Shanghai Moqian Biosciences Co., Ltd, China. The constructed vectors were validated through sequencing by Beijing Tsingke Biotech Co., Ltd, China. Subsequently, the resulting vectors were introduced into *E. coli* BL21(DE3) strain for heterologous expression.

Single colonies were cultured in 100 mL LB medium supplemented with ampicillin (100 mg/L) and grown at 37°C until reaching an optical density of approximately 0.6 at 600 nm. Following pre-cooling for 20 minutes, the 6 × His-tagged fusion protein were induced with 0.1 mM IPTG at 15°C for 20 h. The recombinant proteins were purified using Ni-NTA agarose (Qiagen, Germany), and the target protein was eluted with elution buffer (50 mM NaH_2_PO_4_ pH 8.0, 300 mM NaCl, 250 mM imidazole). The eluate was concentrated using Amicon Ultra-15, PLTK Ultracel-PL 10 kDa (Merck Millipore, Germany). Finally, the purified protein was desalted with storage buffer (50 mM NaH_2_PO_4_ pH 8.0, 20% glycerol) and stored at −80°C. A 10 μL aliquot of the supernatant was taken for SDS-PAGE analysis.

### Enzyme activity assay

The enzymatic activity was conducted according to previously established methods with slight modifications [5]. The biochemical characteristics of GsCGTa, GsCGTb, and GsHIDH were investigated using continuous catalysis in a 50 mM Na_2_HPO_4_-NaH_2_PO_4_ buffer (pH 8.0). GsCGTa initiated the catalytic reaction with UDP-Glc (0.5 mM) as the sugar donor and 2-hydroxynaringenin (0.2 mM) as the receptor at 35°C for 5 min. Subsequently, GsCGTb catalyzed the reaction with UDP-Ara as the sugar donor for 1 h. Following this, an equal volume of GsHIDH was added to the samples and incubated at 35°C for 2 h. The reactions were quenched with an equal volume of methanol and subjected to separation by high-performance liquid chromatography (HPLC). The mobile phase consisted of water containing 0.1% formic acid (v/v, A) and methanol (B) and a gradient elution program was employed as follows: 0–3 min, 27% B; 19 min, 27–65% B; 20 min, 65–60% B; 25 min, 60% B; 29 min, 67% B; 30 min, 95% B. The liquid chromatography column used was InertSustain C18 (4.6 × 150 mm, 5 μm) with a flow rate of 1 mL/min. Detection was performed at wavelengths of 335 nm and 290 nm, with the column temperature maintained at 40°C. Mass spectrometer (MS) analysis was carried out using the Thermo Scientific TSQ Endura LC-ESI-MS system with a UPLC Hypersil Gold column (100 $\times$ 2.1 mm 1.9 μm, Thermo Scientific) at 40°C and a flow rate 0.2 mL/min. The injection volume for analysis was 1 μL. The MS operating parameters were set as follows: ion source temperature 250°C, sheath gas 35 arb, aux gas 10 arb, ion transfer tube temperature 275°C, ion spray voltage 2.5 KV for negative mode, and a mass scan range (m/z) of 200–1000.

### Hairy roots transformation in GSO

To investigate the catalytic activity of GsCGTa and GsCGTb *in vivo*, the full-length cDNAs were cloned into the psuper1300-GFP vector to generate over-expression constructs. These constructs were then individually introduced into *Agrobacterium rhizogenes* K599 strain to induce hairy roots from the hypocotyl of 7-day-old GSO seedlings. Subsequently, the explants were cultured on 1/2 MS solid medium supplemented with 200 mg/L cefotaxime for 2 weeks until hairy roots appeared at the wounded sites. The positive transgenic hairy roots expressing green fluorescent protein (GFP) were identified using fluorescence microscopy and confirmed by both RT-qPCR as well as Western blot analysis. The positive hairy roots were then cultured independently on 1/2 MS solid medium with 200 mg/L cefotaxime for an additional two weeks before being transferred to liquid 1/2 MS medium containing 50 mg/L cefotaxime and incubated at 60 rpm. After 30 days, the hairy roots were harvested, extracted twice with methanol using a sonicator for 20 minutes each, and subjected to HPLC analysis as described in the enzyme activity assay section.

## Acknowledgements

This work was supported partially by grants from Key Area R&D Project of Guangdong Province (2020B020221001), Key Technologies R&D Program of Guangdong Province (2022B1111230001), Guangdong Provincial Key Laboratory of Applied Botany (AB2018017), Youth Innovation Promotion Association CAS (2015286), and Guangdong Provincial Special Fund for Modern Agriculture Industry Technology Innovation Teams, China (2024KJ148).

## Author contributions

S.Z. and Y.W. conceived and supervised the project. S.Z., Z.W., D.S., and G.B. analysed the data. Z.W., D.S., F.Y., and T.L. performed the experiments. T.P., J.Y., and S.Z. discussed and revised the manuscript. S.Z. and Z.W. drafted the manuscript. All the authors reviewed the final manuscript.

## Conflict of interest statement

The authors declare that they have no conflict of interest.

## Data availability

The genome raw sequencing (including PacBio, 10X-Genomics, Hi-C, Illumina) are accessible in the Genome Sequence Archive (GSA) database (https://ngdc.cncb.ac.cn/gsa/) with the accession number CRA013313. Moreover, the assembled genome was deposited in the Figshare (https://figshare.com/).

## Supplementary data

Supplementary data is available at *Horticulture Research* online.

## Supplementary Material

Web_Material_uhae089
